# Microarray analysis of the transcriptional responses of *Porphyromonas gingivalis* to polyphosphate

**DOI:** 10.1186/s12866-014-0218-2

**Published:** 2014-08-24

**Authors:** Ji-Hoi Moon, Jae-Hyung Lee, Jin-Yong Lee

**Affiliations:** 1 Department of Maxillofacial Biomedical Engineering, School of Dentistry, and Institute of Oral Biology, Kyung Hee University, 26 Kyungheedae-ro, Dongdaemun-gu, Seoul 130-701, Republic of Korea; 2 Department of Life and Nanopharmaceutical Sciences, Kyung Hee University, 26 Kyungheedae-ro, Dongdaemun-gu, Seoul 130-701, Republic of Korea

**Keywords:** Porphyromonas gingivalis, Polyphosphate, Transcriptome, Microarray, Gene ontology (GO), Protein-protein interaction network analysis

## Abstract

**Background:**

Polyphosphate (polyP) has bactericidal activity against a gram-negative periodontopathogen *Porphyromonas gingivalis*, a black-pigmented gram-negative anaerobic rod. However, current knowledge about the mode of action of polyP against *P. gingivalis* is incomplete. To elucidate the mechanisms of antibacterial action of polyP against *P. gingivalis*, we performed the full-genome gene expression microarrays, and gene ontology (GO) and protein-protein interaction network analysis of differentially expressed genes (DEGs).

**Results:**

We successfully identified 349 up-regulated genes and 357 down-regulated genes (>1.5-fold, *P* < 0.05) in *P. gingivalis* W83 treated with polyP75 (sodium polyphosphate, Na_n+2_P_n_O_3n+1_; n = 75). Real-time PCR confirmed the up- and down-regulation of some selected genes. GO analysis of the DEGs identified distinct biological themes. Using 202 DEGs belonging to the biological themes, we generated the protein-protein interaction network based on a database of known and predicted protein interactions. The network analysis identified biological meaningful clusters related to hemin acquisition, energy metabolism, cell envelope and cell division, ribosomal proteins, and transposon function.

**Conclusions:**

polyP probably exerts its antibacterial effect through inhibition of hemin acquisition by the bacterium, resulting in severe perturbation of energy metabolism, cell envelope biosynthesis and cell division, and elevated transposition. Further studies will be needed to elucidate the exact mechanism by which polyP induces up-regulation of the genes related to ribosomal proteins. Our results will shed new light on the study of the antibacterial mechanism of polyP against other related bacteria belonging to the black-pigmented *Bacteroides* species.

## Background

Inorganic polyphosphate (polyP) is a chain of few or many hundreds of phosphate (Pi) residues linked by high-energy phosphoanhydride [1]. polyP has attracted considerable attention as a GRAS (generally recognized as safe) food additive by FDA with antimicrobial properties that can prevent spoilage of food [2],[3]. polyP inhibits the growth of various gram-positive bacteria such as *Staphylococcus aureus*[4]-[8], *Listeria monocytogenes*[8],[9], *Sarcina lutea*[7], *Bacillus cereus*[10], and mutans streptococci [11],[12], and of fungi such as *Aspergillus flavus*[5]. The ability of polyP to chelate divalent cations is regarded as relevant to the antibacterial effects, contributing to cell division inhibition and loss of cell-wall integrity [5],[13],[14]. On the other hand, large numbers of gram-negative bacteria including *Escherichia coli* and *Salmonella enterica* serovar Typhimurium are capable of growing in higher concentrations, even up to 10% of polyP [5],[7],[15].

Periodontal disease is caused by bacterial infection which is associated with gram-negative oral anaerobes. In our previous study [16], polyP (Na_n+2_P_n_O_3n+1_; n = the number of phosphorus atoms in the chain) with different linear phosphorus (Pi) chain lengths (3 to 75) demonstrated to have antibacterial activity against *Porphyromonas gingivalis*, a black pigmented, gram-negative periodontopathogen. polyP also showed antibacterial activity against other black-pigmented, gram-negative oral anaerobes such as *Prevotella intermedia* and *Porphyromonas endodontalis*[17],[18]. However, the antimicrobial mechanism of polyP against gram-negative bacteria has not yet been fully understood. In the past decade, global genome-wide studies of changes in expression patterns in response to existing and new antimicrobial agents have provided us with a deeper understanding of antimicrobial action [19]. In the present study, we performed the full-genome gene expression microarrays of *P. gingivalis*, and gene ontology (GO) and protein-protein interaction network analysis of the differentially expressed genes were also performed for elucidating the mechanism of antibacterial action of polyP.

## Results and discussion

**Figure 1:**
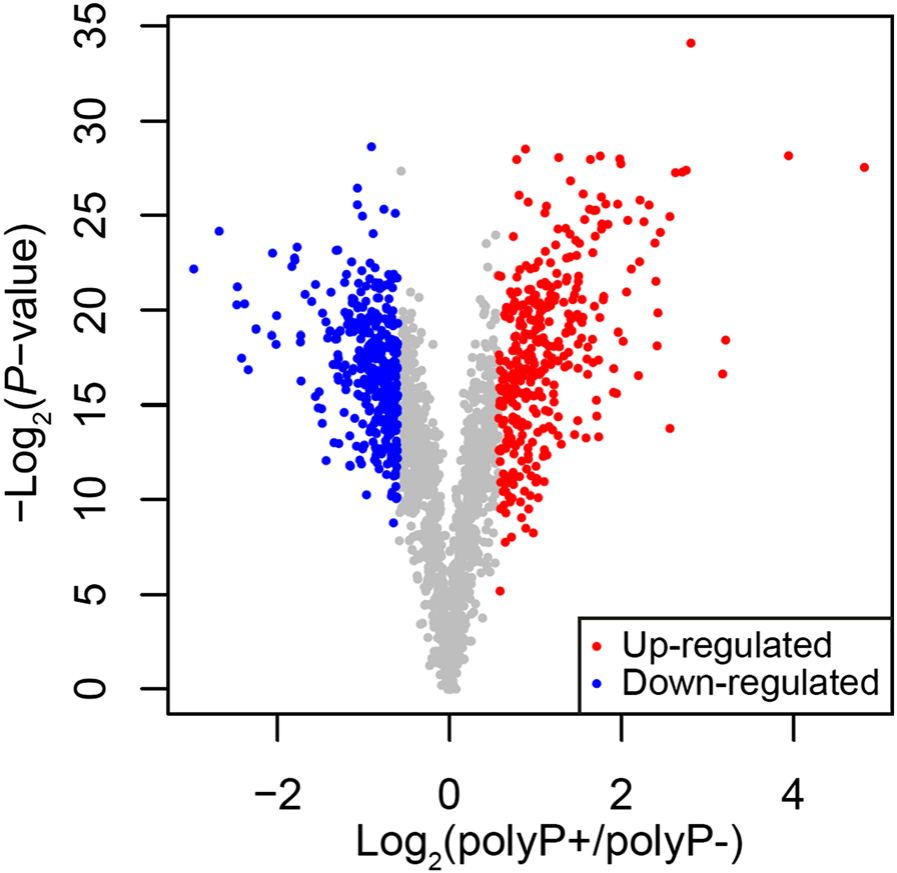
**Differential gene expression in*P. gingivalis*W83 by polyP75 treatment.** Differentially expressed genes with 1.5 fold change and *P*-value < 0.05 were plotted. X-axis presents fold difference between log_2_ expression of polyP75 treatment and no treatment, and y-axis shows the –log_10_*P* -value. Up-regulated genes (over-expressed in polyP75 treatment) were represented as red color and down-regulated genes were colored in blue.

**Figure 2:**
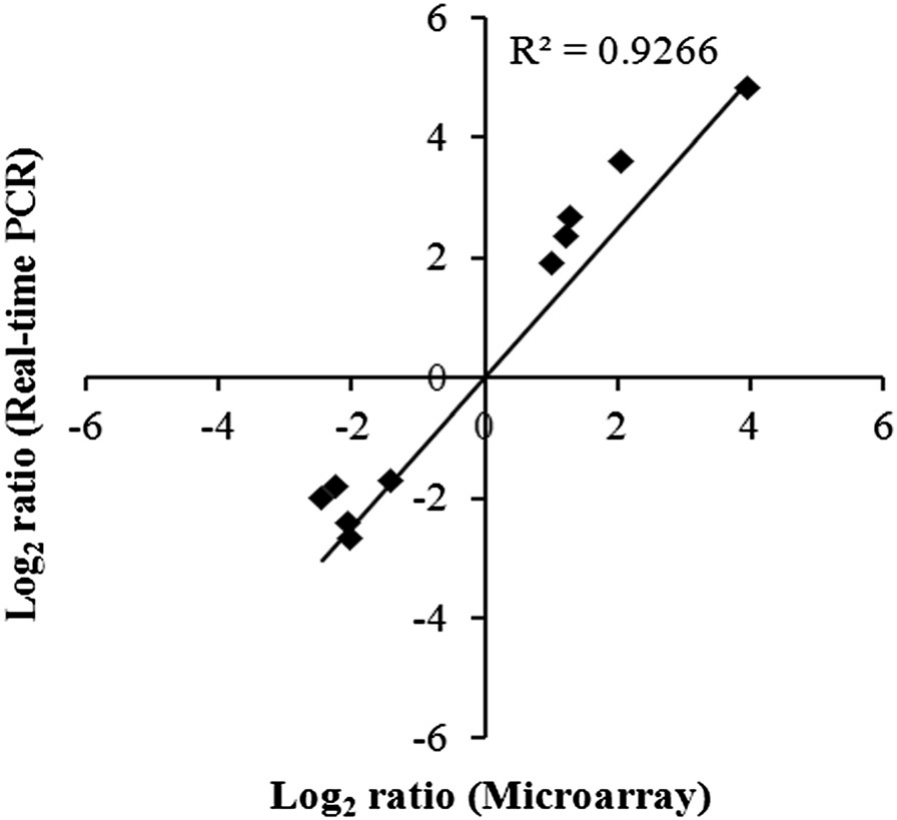
**Comparison of transcription measurements by microarray and qRT-PCR.** The relative transcription levels for 10 genes are listed in Table 6. The qRT-PCR log_2_ values were plotted against the microarray data log_2_ values. The correlation coefficient (*r*) for comparison of the two datasets is 0. 92.

The complete list of the average gene expression values has been deposited in NCBI’s Gene Expression Omnibus (GEO) (http://www.ncbi.nlm.nih.gov/geo/) and is accessible through GEO Series accession number GSE11471. Using filtering criteria of a 1.5 or greater fold-change in expression and significance *P*-values of <0.05, 706 out of 1,909 genes in *P. gingivalis* W83 were differentially expressed by polyP75 treatment. The expression of 349 transcripts was increased by polyP treatment while 357 showed decreased expression (Figure 1). To validate the microarray results, quantitative RT-PCR (qRT-PCR) of selected genes was performed. Five of the genes were selected from the up-regulated group and the other five from the down-regulated group in the polyP-treated *P. gingivalis* cells. We used 16S rRNA as a reference gene for normalization of the qRT-PCR data. There was a high correlation between the expression ratios determined by the microarray and the qRT-PCR (*r* = 0.926) (Figure 2).

To broadly characterize the differentially expressed gene (DEG, up- and down-regulated genes) set, GO category enrichment analysis was performed. This analysis identified distinct biological themes associated with each group of the up-regulated and the down-regulated genes. The down-regulated genes were associated with GO terms related to metabolic process (GO:0008152, *P* = 0.0004), pyridine nucleotide biosynthetic process (GO:0019363, *P* = 0.0012), regulation of cell shape (GO:0008360, *P* = 0.002), and polysaccharide biosynthetic process (GO:0000271, *P* = 0.0015). The up-regulated genes were associated with GO terms related to cellular iron ion homeostasis (GO:0006879, *P* < 0.0001), ribosome (GO:0005840, *P* = 0.0032), transposase activity (GO:0004803, *P* < 0.0001), and DNA binding (GO:0003677, *P* < 0.0001).

**Figure 3:**
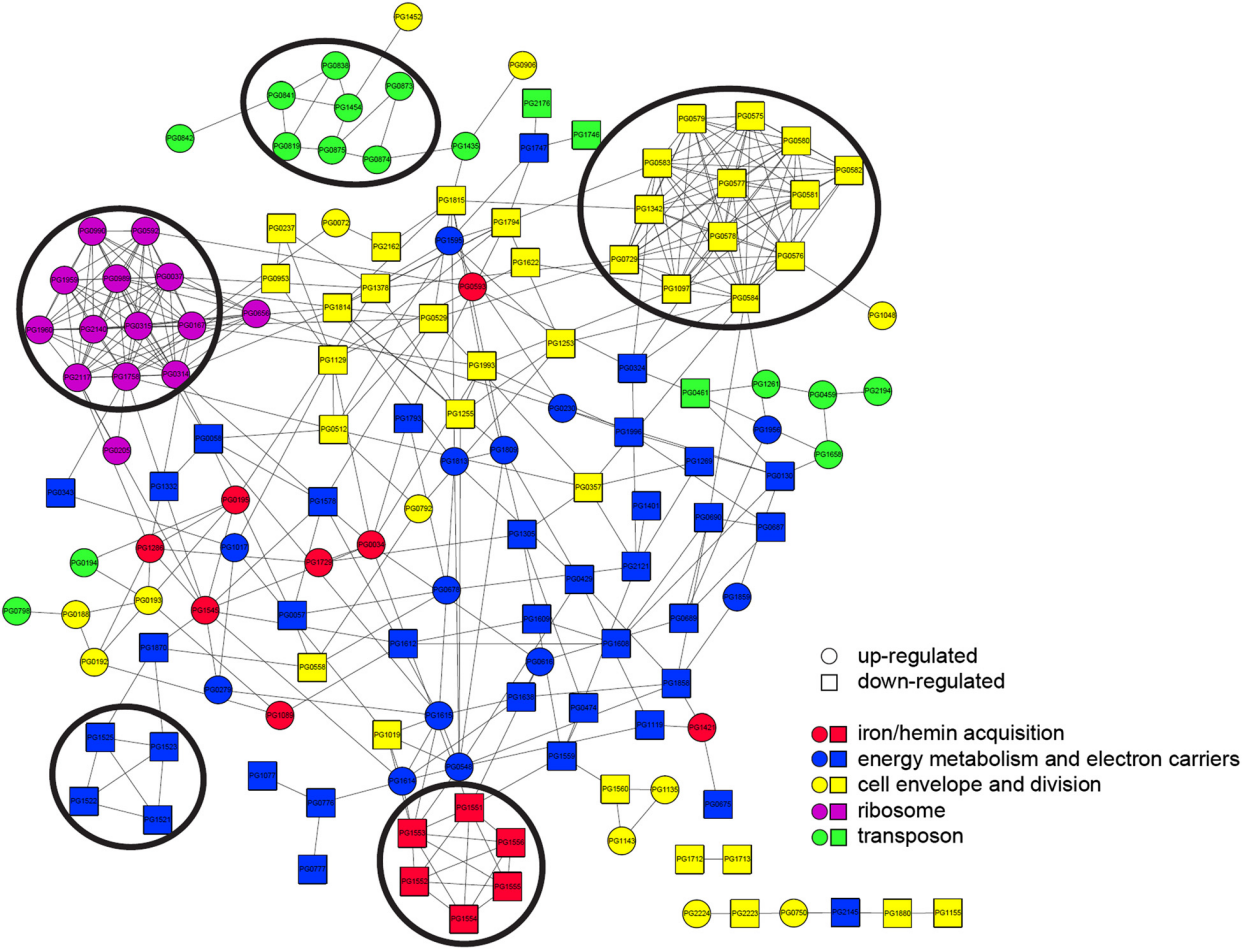
**Protein-protein interaction network of differentially expressed functional genes.** The network was constructed based on the STRING database. Nodes (symbolized as circles and square) and edges (linking lines) represent DEGs and interactions among DEGs, respectively. Up-regulated genes were represented as a circular shape and down-regulated genes were presented as a square shape. Node color represents the functional annotation of each gene. By applying MCODE clustering algorithm, 5 clusters with the score greater than 3 were obtained.

Using 202 DEGs belonging to the above biological themes, we generated the protein-protein interaction network based on a database of known and predicted protein interactions. The network analysis identified 162 DEGs that have direct interaction with one another (Figure 3), and 5 biological meaningful clusters related to 1) iron/hemin acquisition, 2) energy metabolism and electron carriers, 3) cell envelope and cell division, 4) ribosome, and 5) transposon functions.

### Hemin acquisition and energy metabolism

In prokaryotic cells, respiration occurs in the cell membrane in which electrons are transferred sequentially through lipoquinones (menaquinones and ubiquinones) and a series of membrane-bound protein carriers such as cytochrome bc1 complex, although the exact organization of enzymes in the respiratory chains varies among different bacteria [20]. *P. gingivalis* requires hemin as an iron source for its growth [21]. The redox potential of hemin (heme), required as a prosthetic group of cytochrome b, allows it to mediate electron transport with generation of cellular energy [22],[23].

Among 6 genes of *hmu* locus (PG1551 to PG1556) encoding Hmu YRSTUV, which play a major role in hemin acquisition [24], five genes, but not *hmuY*, exhibited more than 2-fold decrease in the expression in the presence of polyP75 (Table 1). In addition, genes related to metabolic process including energy metabolism and biosynthesis of lipoquinones, which occupy a central and essential role in electron transport [20], were significantly down-regulated by polyP (Table 2). Genes related to biosynthesis of pyridine nucleotides, known as soluble electron carriers, were also down-regulated (Table 2). These results are compatible with our previous study in which the amount of hemin accumulated on the *P. gingivalis* surface increased while energy-driven uptake of hemin by the bacterium decreased in the presence of polyP75 [16]. It is conceivable that polyP induce hemin deficiency in *P. gingivalis*, resulting in disruption of the electron transport occurring in the bacterial membrane. Notably, the up-regulation of oxidative stress response was observed under hemin-limited conditions [25]. Hence, the up-regulation of a series of genes involved in oxidative stress, i.e., 4Fe-4S ferredoxin, rubrerythrin, thioredoxin, Fe-Mn superoxide dismutase, thiol peroxidase, Dps family protein, RprY, ferritin, and HtrA (Table 1), may be due to hemin limitation induced by polyP. However, it is also possible that excessive accumulation of hemin in the vicinity of the bacterial cell surface without formation of μ-oxo bisheme by the bacterium may cause oxidative stress on *P. gingivalis*[16], as the formation of μ-oxo bisheme protects from hemin-mediated cell damage [23],[26],[27].

**Table 1:** Differentially expressed genes related to iron/hemin aquisition and oxidative stress

**Locus no.**^**a**^	**Putative identification**^**a**^	**Cellular role**^**a**^	**Avg fold difference**^**b**^
PG1551	hmuY protein	Transport and binding proteins: Cations and iron carrying compounds	−1.19^c^
PG1552	TonB-dependent receptor HmuR	Transport and binding proteins: Cations and iron carrying compounds	−2.28
PG1553	HmuS^d^	Hemin acquisition^d^	−2.77
PG1554	HmuT^d^	Hemin acquisition^d^	−3.44
PG1555	HmuU^d^	Hemin acquisition^d^	−3.29
PG1556	HmuV^d^	Hemin acquisition^d^	−2.15
PG1729	thiol peroxidase	Cellular processes : Detoxification	3.12
PG1421	Ferredoxin, 4Fe-4S	Energy metabolism : Electron transport	28.54
PG0195	Rubrerythrin	Energy metabolism : Electron transport	15.49
PG0034	Thioredoxin	Energy metabolism : Electron transport	2.76
PG1286	Ferritin	Transport and binding proteins:	2.59
		Cations and iron carrying compounds	
PG0090	Dps family protein	Cellular processes:	2.45
		Adaptations to atypical conditions	
PG1545	Superoxide dismutase, Fe-Mn	Cellular processes : Detoxification	2.34
PG1089	DNA-binding response regulator RprY	Regulatory functions : DNA interactions	2.00
		Signal transduction: Two-component systems	
PG0593	htrA protein heat induced serine protease	Protein fate: Degradation of proteins, peptides, and glycopeptides	4.20

^a^Locus number, putative identification, and cellular role are according to the TIGR genome database.

^b^Average fold difference indicates the expression of the gene by polyP addition versus no polyP addition.

^c^The cut off ratio for the fold difference was < 1.5.

^d^Putative identification and cellular role are according to Lewis [24].

**Table 2:** Differentially expressed genes related to energy metabolism and biosynthesis of electron carriers

**Locus no.**^**a**^	**Putative identification**^**a**^	**Avg fold difference**^**b**^
Energy metabolism : Amino acids and amines		
PG1269	Delta-1-pyrroline-5-carboxylate dehydrogenase	−2.02
PG0474	Low-specificity L-threonine aldolase	−1.93
PG1401	Beta-eliminating lyase	−1.74
PG0343	Methionine gamma-lyase	−1.64
PG1559	Aminomethyltransferase	−1.54
PG0324	Histidine ammonia-lyase	−1.53
PG1305	Glycine dehydrogenase	−1.52
PG2121	L-asparaginase	−1.51
PG0025	Fumarylacetoacetate hydrolase family protein	2.11
Energy metabolism : Anaerobic/Fermentation		
PG0687	Succinate-semialdehyde dehydrogenase	−1.76
PG0690	4-hydroxybutyrate CoA-transferase	−1.66
PG0689	NAD-dependent 4-hydroxybutyrate dehydrogenase	−1.58
PG1609	Methylmalonyl-CoA decarboxylase, gamma subunit	−1.87
PG1612	Methylmalonyl-CoA decarboxylase, alpha subunit	−1.71
PG1608	Methylmalonyl-CoA decarboxylase, beta subunit	−1.64
PG0675	Indolepyruvate ferredoxin oxidoreductase, alpha subunit	−1.53
PG1809	2-oxoglutarate oxidoreductase, gamma subunit	2.18
PG1956	4-hydroxybutyrate CoA-transferase	1.74
Energy metabolism : Biosynthesis and degradation of polysaccharides		
PG2145	Polysaccharide deacetylase	−1.94
PG0897	Alpha-amylase family protein	−1.85
PG1793	1,4-alpha-glucan branching enzyme	−1.67
Energy metabolism : Electron transport		
PG0776	Electron transfer flavoprotein, alpha subunit	−2.30
PG0777	Electron transfer flavoprotein, beta subunit	−1.91
PG1638	Thioredoxin family protein	−1.88
PG1332	NAD(P) transhydrogenase, beta subunit	−1.83
PG1119	Flavodoxin, putative	−1.69
PG0429	Pyruvate synthase	−1.64
PG1077	Electron transfer flavoprotein, beta subunit	−1.57
PG1858	Flavodoxin	−2.57
PG2178	NADH:ubiquinone oxidoreductase, Na translocating, E subunit	−1.51
PG0034	Thioredoxin	2.76
PG0195	Rubrerythrin	15.49
PG0548	Pyruvate ferredoxin/flavodoxin oxidoreductase family protein	2.58
PG0616	Thioredoxin, putative	1.52
PG1421	Ferredoxin, 4Fe-4S	28.54
PG1813	Ferredoxin, 4Fe-4S	1.65
Energy metabolism : Glycolysis/gluconeogenesis		
PG0130	Phosphoglyceromutase	−1.68
Energy metabolism : Purines, pyrimidines, nucleosides, and nucleotides		
PG1996	Deoxyribose-phosphate aldolase	−1.73
Energy metabolism : Pentose phosphate pathway		
PG1747	Ribose 5-phosphate isomerase B, putative	−2.45
PG0230	Transaldolase	2.05
PG1595	Ribulose-phosphate 3-epimerase	2.22
Energy metabolism: Sugars		
PG1633	Galactokinase	−1.89
Energy metabolism : TCA cycle		
PG1614	Succinate dehydrogenase	2.25
PG1615	Succinate dehydrogenase	1.60
Energy metabolism : Other		
PG1522	Mandelate racemase/muconate lactonizing enzyme family protein	−2.24
PG0279	NADP-dependent malic enzyme	1.82
PG1017	Pyruvate phosphate dikinase	1.75
PG1513	Phosphoribosyltransferase, putative/phosphoglycerate mutase family protein	3.05
PG1859	Glycerate kinase family protein	1.76
Biosynthesis of pyridine nucleotides		
PG0058	Nicotinic acid mononucleotide adenyltransferase	−1.93
PG1578	Quinolinate synthetase	−1.62
PG0057	Nicotinate phosphoribosyltransferase	−1.61
PG0678	Pyrazinamidase/nicotinamidase, putative	2.00
Biosynthesis of menaquinone and ubiquinone		
PG1870	Methlytransferase, UbiE/COQ5 family	−2.60
PG1467	Methlytransferase, UbiE/COQ5 family	−2.46
PG1523	Naphthoate synthase	−1.89
PG1521	O-succinylbenzoic acid--CoA ligase	−1.78
PG1525	Isochorismate synthase, putative	−1.50

^a^Locus number, putative identification, and cellular role are according to the TIGR genome database.

^b^Average fold difference indicates the expression of the gene by polyP addition versus no polyP addition.

### Cell envelope and cell division

Among genes involved in biosynthesis and degradation of surface polysaccharides and lipopolysaccharides, 9 genes were repressed and 5 genes increased by polyP. Among genes related to biosynthesis and degradation of murein sacculus and peptidoglycan, 7 genes were down-regulated (Table 3). For most bacteria, the peptidoglycan cell wall is both necessary and sufficient to determine cell shape [28]. In *P. gingivalis* W83 genome there is a group of genes called division/cell wall (DCW) cluster, which are involved in cell division and synthesis of peptidoglycan [29]-[31]: PG0575 (penicillin-binding protein 2), PG0576 (*murE*), PG0577 (*mraY*), PG0578 (*murD*), PG0579 (*ftsW*), PG0580 (*murG*), PG0581 (*murC*), PG0582 (*ftsQ*), PG0583 (*ftsA*), and PG0584 (*ftsZ*). Among these, *mraY*, *murD*, *ftsW*, *murG*, *murC*, and *ftsQ* (PG0577- PG0582) were down-regulated by polyP75. It seems that the reduced expression of the genes related to cell envelope biosynthesis in polyP-exposed *P. gingivalis* may be a result from disruption of the electron transport and reduced production of ATP, since ATP is fundamental for many metabolic processes in bacteria including cell wall biosynthesis and protein synthesis [32]. These transcriptional changes are partially in agreement with the previous report using *Bacillus cereus* in which polyP inhibited the bacterial cell division [10]. However, unlike *B. cereus*, formation of elongated aseptate cells and growth phase-dependent bacteriolysis were not observed in *P. gingivalis* exposed to polyP [16]. It was proposed that polyP, because of its metal ion-chelating nature, may affect the ubiquitous bacterial cell division protein FtsZ, whose GTPase activity is known to be strictly dependent on divalent metal ions. Then, polyP may consequently block the dynamic formation (polymerization) of the Z ring, which would explain the aseptate phenotype of *B. cereus*[10]. *B. cereus* exposed to polyP, however, showed normal DNA replication, chromosome segregation, and synthesis of the lateral cell wall [10]. In the present study, *P. gingivalis* W83 decreased the expression of genes in relation to biosynthesis of cell wall, purine, pyrimidine, nucleoside, and nucleotide, and replication of DNA in the presence of polyP75 (Table 3). These results probably indicate that polyP affects the overall proliferation process including biosynthesis of nucleic acids, DNA replication, biosynthesis of cell wall, and cell division in *P. gingivalis*.

**Table 3:** Differentially expressed genes related to cell envelope and cell division

**Locus no.**^**a**^	**Putative identification**^**a**^	**Avg fold difference**^**b**^
Cell envelope : Biosynthesis and degradation of murein sacculus and peptidoglycan		
PG0575	Penicillin-binding protein 2	−1.41^c^
PG0576	UDP-N-acetylmuramoylalanyl-D-glutamyl-2, 6-diaminopimelate ligase	−1.42^c^
PG0577	Phospho-N-acetylmuramoyl-pentapeptide-transferase	−1.56
PG0578	UDP-N-acetylmuramoylalanine--D-glutamateligase	−1.58
PG0580	N-acetylglucosaminyl transferase	−1.78
PG0581	UDP-N-acetylmuramate--L-alanine ligase	−1.81
PG1342	UDP-N-acetylenolpyruvoylglucosamine reductase	−2.17
PG0729	D-alanylalanine synthetase	−1.80
PG1097	Mur ligase domain protein/alanine racemase	−1.58
Cellular process: Cell division		
PG0579	Cell division protein FtsW	−1.74
PG0582	Cell division protein FtsQ	−1.80
PG0583	Cell division protein FtsA	−1.32 ^c^
PG0584	Cell division protein FtsZ	−1.36 ^c^
Cell envelope : Biosynthesis and degradation of surface polysaccharides and lipopolysaccharides		
PG1155	ADP-heptose--LPS heptosyltransferase, putative	−1.94
PG1783	Glycosyl transferase, group 2 family protein	−1.87
PG2223	Glycosyl transferase, group 2 family protein	−1.77
PG1815	3-deoxy-manno-octulosonate cytidylyltransferase	−1.73
PG1712	Alpha-1,2-mannosidase family protein	−1.69
PG1345	Glycosyl transferase, group 1 family protein	−1.66
PG2162	Lipid A disaccharide synthase	−1.65
PG1560	dTDP-glucose 4,6-dehydratase	−1.57
PG1880	Glycosyl transferase, group 2 family protein	−1.53
PG0072	UDP-3-O-[3-hydroxymyristoyl] glucosamine N-acyltransferase	1.83
PG0750	Glycosyl transferase, group 2 family protein	1.51
PG1048	N-acetylmuramoyl-L-alanine amidase, family 3	2.96
PG1135	Bacterial sugar transferase	5.28
PG1143	Sugar dehydrogenase, UD-glucose/GDP-mannose dehydrogenase family	1.89
Cell envelope : Other		
PG1019	Lipoprotein, putative	−5.47
PG1180	Hypothetical protein	−4.15
PG1713	Lipoprotein, putative	−2.01
PG1767	Lipoprotein, putative	−1.96
PG0490	Hypothetical protein	−1.74
PG1005	Lipoprotein, putative	−1.65
PG1948	Lipoprotein, putative	−1.56
PG0670	Lipoprotein, putative	−1.54
PG2155	Lipoprotein, putative	−1.53
PG1600	Hypothetical protein	−1.52
PG0188	Lipoprotein, putative	1.66
PG0192	Cationic outer membrane protein OmpH	2.68
PG0193	Cationic outer membrane protein OmpH	2.18
PG0717	Lipoprotein, putative	1.95
PG0906	Lipoprotein, putative	1.94
PG1452	Lipoprotein, putative	1.52
PG1828	Lipoprotein, putative	1.87
PG2105	Lipoprotein, putative	1.98
PG2224	Hypothetical protein	2.19
DNA metabolism : DNA replication, recombination, and repair		
PG1814	DNA primase	−2.01
PG1993	Excinuclease ABC, C subunit	−1.77
PG1255	Recombination protein RecR	−1.64
PG1253	DNA ligase, NAD-dependent	−1.62
PG0237	Uracil-DNA glycosylase	−1.58
PG1378	A/G-specific adenine glycosylase	−2.83
PG1622	DNA topoisomerase IV subunit A	−2.02
PG1794	DNA polymerase type I	−1.51
PG2009	DNA repair protein RecO, putative	2.34
Purines, pyrimidines, nucleosides, and nucleotides : 2′-Deoxyribonucleotide metabolism		
PG1129	Ribonucleotide reductase	−2.30
PG0953	Deoxyuridine 5′-triphosphate nucleotidohydrolase	−2.14
Purines, pyrimidines, nucleosides, and nucleotides : Nucleotide and nucleoside interconversions		
PG0512	Guanylate kinase	−1.89
Purines, pyrimidines, nucleosides, and nucleotides : Pyrimidine ribonucleotide biosynthesis		
PG0529	Carbamoyl-phosphate synthase small subunit	−1.70
PG0357	Aspartate carbamoyltransferase catalytic subunit	−1.54
Purines, pyrimidines, nucleosides, and nucleotides : Salvage of nucleosides and nucleotides		
PG0558	Purine nucleoside phosphorylase	−1.51
PG0792	Hypoxanthine phosphoribosyltransferase	2.25

^a^Locus number, putative identification, and cellular role are according to the TIGR genome database.

^b^Average fold difference indicates the expression of the gene by polyP addition versus no polyP addition.

^c^The cut off ratio for the fold difference was < 1.5.

In several transcriptional profiling studies using gram-positive bacteria, a cell wall stress stimulon that includes genes involved in peptidoglycan biosynthesis was induced in the cells challenged with cell wall-active antibiotics [33],[34]. The bacterial cells appeared to respond to the cell wall-active antibiotics by attempting to raise the rate of peptidoglycan biosynthesis in order to compensate for the damaged and partially missing cell wall [35],[36]. Overall, the results indicate that the mode of action of polyP against *P. gingivalis* may be different from not only that of the cell wall-active antibiotics against gram-positive bacteria, but also that of polyP against gram-positive bacteria.

### Ribosomal proteins

In bacteria, production of ribosome requires up to 40% of the cell's energy in rapidly growing bacteria and is therefore tightly regulated on several levels [37]. It seems that bacteria with kinetically impaired ribosomes can to some extent increase the number of ribosomes accumulated under poor growth conditions or under antibiotic challenge in order to compensate for their slower function [38],[39]. It has been reported that antibiotics that target the ribosome or translation factors up-regulate synthesis of ribosomal proteins and accumulate ribosome precursors in *Streptococcus pneumoniae*[40]. Similarly, in *Clostridium difficile*, genes encoding many ribosomal proteins were coordinately up-regulated by antibiotics such as amoxicillin, clindamycin, and metronidazole [38]. Therefore, it is conceivable that the up-regulation of the genes encoding ribosomal proteins of polyP- exposed *P. gingivalis* (Table 4) may reflect a compensatory response for slower or disturbed function of the ribosome.

**Table 4:** Differentially expressed genes related to ribosome

**Locus no.**^**a**^	**Putative identification**^**a**^	**Avg fold difference**^**b**^
Protein synthesis : Ribosomal proteins		
PG0037	50S ribosomal protein L19	3.23
PG0167	Ribosomal protein L25	1.86
PG0314	Ribosomal protein L21	1.90
PG0315	50S ribosomal protein L27	1.78
PG0385	Ribosomal protein S21	3.98
PG0592	50S ribosomal protein L31	4.01
PG0656	50S ribosomal protein L34	6.80
PG0989	50S ribosomal protein L20	3.43
PG0990	Ribosomal protein L35	1.74
PG1723	Ribosomal protein S20	2.94
PG1758	Ribosomal protein S15	6.23
PG1959	Ribosomal protein L33	2.02
PG1960	Ribosomal protein L28	2.03
PG2117	30S ribosomal protein S16	2.93
PG2140	Ribosomal protein L32	3.40
PG0205	Peptide chain release factor 3	1.50

^a^Locus number, putative identification, and cellular role are according to the TIGR genome database.

^b^Average fold difference indicates the expression of the gene by polyP addition versus no polyP addition.

Meanwhile, ribosome biosynthesis of bacteria is governed by transcriptional and translational regulatory mechanisms that provide a balanced and coordinated production of individual ribosomal components [41]. It has been suggested that some free ribosomal proteins act as autogenous feedback inhibitors that cause selective translational inhibition of the synthesis of certain ribosomal proteins whose genes are in the same operon as their own. This inhibition is due to the structural homology between certain ribosomal protein binding regions on 16S rRNA and the mRNA target site for the ribosomal protein [42]-[44]. Although autogenous regulation is known to be a general strategy of balancing ribosomal protein synthesis in bacteria [41], mechanisms for controlling ribosomal protein gene expression in *P. gingivalis* have not yet been characterized. Further studies will be needed to elucidate the regulatory mechanisms involved in ribosomal protein synthesis in *P. gingivalis*.

### Transposon functions

The majority of the up-regulated genes related to mobile and extrachromosomal element functions were the genes encoding transposases (Table 5). Transposition is generally known to be triggered by cellular stress, i.e., nutritional deficiency, chemicals, and oxidative agents. Little is known about the transposition in *P. gingivalis*, but up-regulation of transposase-related insertion sequence elements was noticed in *P. gingivalis* W50 after treatment with H_2_O_2_[45]. Thus, it seems quite reasonable to speculate that induction of transposase is associated with oxidative stress-like response which occurred in *P. gingivalis* W83 due to the presence of polyP.

**Table 5:** Differentially expressed genes related to transposon functions

**Locus no.**	**Putative identification**	**Avg fold difference**
Mobile and extrachromosomal element functions: Transposon functions		
PG0019	ISPg4 transposase	1.57
PG0050	ISPg4, transposase	1.81
PG0177	ISPg4, transposase	1.87
PG0194	ISPg3, transposase	2.18
PG0225	ISPg4, transposase	1.80
PG0261	ISPg3, transposase	2.20
PG0459	ISPg5, transposase	1.60
PG0487	ISPg4, transposase	1.98
PG0798	ISPg3, transposase	2.11
PG0819	Integrase	1.80
PG0838	Integrase	3.36
PG0841	Mobilizable transposon, excision protein, putative	3.78
PG0842	Mobilizable transposon, hypothetical protein, putative	2.84
PG0872	Mobilizable transposon, xis protein	3.87
PG0873	Mobilizable transposon, tnpC protein	9.34
PG0874	Mobilizable transposon, int protein	2.42
PG0875	Mobilizable transposon, tnpA protein	1.68
PG0970	ISPg4, transposase	1.79
PG1032	ISPg3, transposase	2.23
PG1061	ISPg6, transposase	2.03
PG1261	ISPg4, transposase	2.06
PG1262	ISPg3, transposase	2.11
PG1435	Integrase	2.77
PG1454	Integrase	1.88
PG1658	ISPg4, transposase	1.83
PG1673	ISPg4, transposase	1.77
PG2194	ISPg4, transposase	1.85
PG0461	ISPg7, transposase	−2.77
PG0277	ISPg2, transposase	−1.58
PG0865	ISPg2, transposase	−1.53
PG1746	ISPg2, transposase	−1.63
PG2176	ISPg2, transposase	−1.58
PG1350	ISPg2, transposase	−1.53

## Conclusions

We observed that polyP causes numerous events of differential transcription in *P. gingivalis*. Down-regulated genes were related to iron/hemin acquisition, energy metabolism and electron carriers, and cell envelope and cell division. In contrast, up-regulated genes were related to ribosome and transposon functions. polyP probably exerts its antibacterial effect through inhibition of iron/hemin acquisition by the bacterium, resulting in severe perturbation of energy metabolism, cell envelope biosynthesis and cell division, and elevated transposition. Although the up-regulation of the genes related to ribosomal proteins may possibly reflect autogenous feedback inhibition to regulate the synthesis of certain ribosomal proteins in metabolically disturbed *P. gingivalis* by polyP, the exact mechanisms underlying this polyP-induced up-regulation of the genes have yet to be elucidated. The current information obtained from the gene ontology and protein-protein interaction network analysis of the differentially expressed genes determined by microarray will shed new light on the study of the antibacterial mechanism of polyP against other related bacteria belonging to the black-pigmented *Bacteroides* species.

## Methods

### Chemicals

polyP with a chain length of 75 (polyP75; sodium polyphosphate, glassy, Na_n+2_P_n_O_3n+1_; n = 75) was purchased from Sigma Chemical Co. (St. Louis, MO), dissolved in distilled water to a concentration of 10% (wt/vol), sterilized using a 0.22-μm filter, and stored at −20°C until use.

### Bacterial strain and growth conditions

*P. gingivalis* strain W83 (kindly supplied by Dr. Koji Nakayama, Nagasaki University Graduate School of Biomedical Sciences) was cultured at 37°C anaerobically (85% N_2_, 10% H_2_, and 5% CO_2_) in half-strength brain heart infusion (BHI) broth (Becton Dickinson, Sparks, MD) supplemented with 0.5% yeast extract (Difco Laboratories, Detroit, MI), 5 μg/ml of hemin (Sigma), and 1 μg/ml of vitamin K_1_ (Sigma).

### RNA isolation and cDNA synthesis

Use of high concentrations of antibacterial agents for extended periods of time changes the expression of a large set of genes and the effect may be secondary to the action of the drug [46]. Meanwhile, at sub-lethal concentrations, bacteria may sense antibiotics as extracellular chemicals to trigger different cellular responses such as an altered antibiotic resistance/tolerance profile [47]. Hence, we performed the full-genome gene expression microarrays of *P. gingivalis* W83 exposed to polyP75 at a concentration of 0.03%, which was previously determined to be MIC against the bacterium [16], for a short period of time. *P. gingivalis* culture grown to early exponential phase (OD_600_ = 0.3) was divided in half. One aliquot was left untreated, while the other one was treated with 0.03% polyP75. After incubation of both the bacterial cultures for 2 h under anaerobic conditions, the bacterial cells were harvested, and total RNA was extracted from the cells using Trizol Reagent (Invitrogen, Carlsbad, CA). RNA quality was monitored by Agilent 2100 Bioanalyzer (Agilent Technologies, Santa Clara, CA), and RNA quantity was measured by spectrophotometer. All the samples used in this study exhibited A260/A280 ratio of at least 1.8. cDNA was synthesized with 20 μg of total RNA using SuperScript® II Reverse Transcriptase (Invitrogen).

### Microarray analysis

Two individual Cy3-labeled cDNA samples were hybridized into DNA microarrays (Nimblegen Systems, Inc., Madison, WI) containing the whole genome of 1,909 genes of *P. gingivalis* W83 for 16 h at 42°C. Five replicates of the genome were included per chip. An average of 19 different 60-mer probes which had at least three mismatches compared to other 60-mers represented each gene in the genome. A quality control check (hybridization) was performed for each array, which contained on-chip control oligonucleotides. Data were extracted from the scanned images using an Axon GenePix 4000B microarray scanner and NimbleScan Version 2.3. Quantile normalization was performed across replicate arrays, and RMA (Robust Multichip Average) analysis was performed to generate gene expression values. Genes evidencing statistically significant changes in expression (>1.5-fold difference) were identified via t-tests (*P* < 0.05).

### Assessment of array data quality

To confirm the microarray results using qRT-PCR, 10 genes were selected, and specific primers for the selected genes (Table 6) were designed using Primer3 (http://fokker.wi.mit.edu/primer3/). All quantifications were normalized to the *P. gingivalis* 16S rRNA gene. The transcriptional ratio from qRT-PCR analysis was logarithm-transformed and then plotted against the average log_2_ ratio values obtained by microarray analysis [48].

**Table 6:** Real-time quantitative RT-PCR confirmation of selected genes

**Locus no.**^**a**^	**Primer sequence (5′-3′)**^**b**^	**Product size (bp)**
16S rRNA	F: TGTTACAATGGGAGGGACAAAGGG	118
	R: TTACTAGCGAATCCAGCTTCACGG	
PG0090	F: CAGAAGTGAAGGAAGAGCACGAAC	197
	R: GTAGGCAGACAGCATCCAAACG	
PG0195	F: TCCACGGCTGAGAACTTGCG	149
	R: TGCTCGGCTTCCACCTTTGC	
PG1545	F: CCAAACCCTCAACCACAATC	142
	R: GGTACCGGCTGTGTTGAACT	
PG0593	F: CGTGTGGGAGAGTGGGTATTGG	175
	R: CGCCGCTGTTGCCTGAATTG	
PG1089	F: CCATCGCGATCGATGATCAGGTAA	104
	R: GGCATAGTTGCGTTCAAGGGTTTC	
PG1019	F: TTCGCAGTATCCCATCCAAC	126
	R: TCCGGCTCATAGACTTCCAA	
PG1180	F: CAGTCTGCCACAGTTCACCA	124
	R: CCCTACACGGACACTACCGA	
PG1983	F: GCTCTGTGGTGTGGGCTATC	146
	R: GGATAACAGGCAAACCCGAT	
PG0885	F: CAGATCCAAATCGGGACTGA	156
	R: GTAGAGCAAGCCATGCAAGC	
PG1181	F: GATGAATTCGGGCGGATAAT	184
	R: CCTTGAAGTGCTCCAACGAC	

^a^Based on the genome annotation provided by TIGR (http://cmr.jcvi.org/cgi-bin/CMR/GenomePage.cgi?org=gpg).

^b^Primers were designed using Primer3 program for the study except for the primers of *P. gingivalis* 16S rRNA and PG1089 [49], which were prepared based on the primer sequences published previously. The 16S rRNA gene was used as the reference gene for normalization. F, forward; R, reverse.

### Gene ontology (GO) enrichment analysis

The GO term annotations for *P. gingivalis* were downloaded from the Gene Ontology website (http://www.geneontology.org/GO.downloads.annotations.shtml, UniProt [multispecies] GO Annotations @ EBI, Apr. 2013). To test the GO category enrichment, we calculated the fraction of gene in the test set (*F*_*test*_) associated with each GO category. Then, we generated the random control gene set that has the same number gene of test set. In this process, the random control gene was selected by matching the length of the test gene. The fraction of genes in this randomly selected control set (*F*_*control*_) associated with the current GO category was calculated. This random sampling process was repeated 10,000 times. Finally, the *P*-value for the enriched GO category in a test gene set was calculated as the fraction of times that *F*_*test*_ was lower than or equal to *F*_*control*_.

### Protein-protein interaction network analysis

The protein-protein interaction network data including score were obtained from the STRING 9.1 (http://string-db.org) [50], for *P. gingivalis* W83. We used Cytoscape software [51] for network drawing, in which nodes and edges represented DEGs and interactions among DEGs, respectively. DEGs with no direct interaction were discarded, and the final dataset consisting of 611 DEGs and 1,641 interactions were used for the network construction. In order to find significant interaction between DEGs, we applied the confidence cutoff as 0.400 (medium confidence).

To understand the biological functions of the DEGs in the network, we annotated 202 DEGs belonging to 8 relevant biological functional clusters and then generated the sub-network using these DEGs in the whole DEGs network constructed above. Cytoscape plug-in MCODE [52] was used to decompose the sub-network and 5 clusters with the score greater than 3 were identified.

## Abbreviations

polyP: Inorganic polyphosphate

GO: Gene ontology

DEG(s): Differentially expressed genes (s)

qRT-PCR: Quantitative RT-PCR

## Competing interests

The authors declare that they have no competing interests.

## Authors’ contributions

Conceived and designed the experiments: JHM and JYL. Performed the experiments: JHM. Analyzed the data: JHM, JHL. Wrote the manuscript: JHM, JHL and JYL. All authors read and approve the final manuscript.
